# The structure of the Canadian packaged food and non-alcoholic beverage manufacturing and grocery retailing sectors through a public health lens

**DOI:** 10.1186/s12992-023-00917-w

**Published:** 2023-03-11

**Authors:** Alexa Gaucher-Holm, Benjamin Wood, Gary Sacks, Lana Vanderlee

**Affiliations:** 1 École de nutrition, Centre Nutrition, santé et société (NUTRISS), Institut sur la nutrition et les aliments fonctionnels (INAF), Université Laval, 2425 rue de l’Agriculture, Québec, QC G1V 0A6 Canada; 2 Global Centre for Preventive Health and Nutrition (GLOBE), Institute for Health Transformation, Deakin University, 221 Burwood Highway, Burwood, VIC 3125 Australia

**Keywords:** Food environments, Corporate determinants of health, Food industry, Beverage industry, Grocery retail, Market structure, Market concentration, Canada

## Abstract

**Background:**

Corporate power has been recognized as an important influence on food environments and population health more broadly. Understanding the structure of national food and beverage markets can provide important insight into the power held by leading corporations. This study aimed to descriptively analyze the structure of the Canadian food and beverage manufacturing and grocery retailing sectors as of 2020/21.

**Methods:**

Packaged food manufacturers, non-alcoholic beverage manufacturers and grocery retailers with ≥ 1% market share in 2020/21 in Canada as per Euromonitor International were identified and characterized. Proportion of market share held by public vs private, multinational vs national, and foreign multinational companies was assessed for the 3 sectors. The concentration of 14 packaged food, 8 non-alcoholic beverage and 5 grocery retailing markets was assessed using the Herfindahl–Hirschman Index (HHI) and the four firm concentration ratio (CR4) (HHI > 1800 and CR4 > 60 suggest high market concentration). Company ownership structure was also assessed, including common ownership of public companies by three of the largest global asset managers using data from Refinitiv Eikon, a financial market database.

**Results:**

The Canadian non-alcoholic beverage manufacturing sector, and, to a lesser extent, the packaged food manufacturing sector were dominated by foreign multinational companies, in contrast with the grocery retailing sector which was dominated by national companies. Market concentration varied across sectors and markets but was substantially greater within the retailing (median CR4 = 84; median HHI = 2405) and non-alcoholic beverage sectors (median CR4 = 72; median HHI = 1995) compared to the packaged food sector (median CR4 = 51; median HHI = 932). There was considerable evidence of common ownership across sectors. Overall, the Vanguard Group Inc owned at least 1% of shares in 95% of publicly listed companies, Blackrock Institutional Trust Company 71%, and State Street Global Advisors (US) 43%.

**Conclusions:**

The Canadian packaged food and non-alcoholic beverage manufacturing and grocery retailing sectors include several consolidated markets, with a high degree of common ownership by major investors. Findings suggest that a small number of large corporations, particularly in the retailing sector, have extensive power to influence Canadian food environments; their policies and practices warrant substantial attention as part of efforts to improve population diets in Canada.

**Supplementary Information:**

The online version contains supplementary material available at 10.1186/s12992-023-00917-w.

## Introduction

The global rise in obesity and associated non-communicable diseases (NCDs) has coincided with important shifts in global food systems and environments, including changes to the food supply, distribution systems and marketing pathways [1]. Current food systems are unsustainable for both human and planetary health, and their transformation will require substantial change from governments, food companies, and other stakeholders including institutional investors and civil society groups [2, 3].

Food and beverage industries have an important influence on the healthfulness of food environments through the production, pricing, and marketing of their products, as well as their corporate political practices. A substantial amount of public health research has demonstrated the use of corporate strategies, such as the pervasive use of marketing tactics targeting children and adolescents that undermine health [4, 5] and the intense political lobbying used by corporations to strategically shape food policies in their favor [6–8]. More recently, the dominance of major food and beverage companies within global markets has garnered increasing interest from public health scholars, with greater attention being paid to ways in which excessive market power of large food companies may influence the characteristics of food environments and population health, through practices such as mergers and acquisitions, licensing arrangements, and research and development practices [9–11].

It is increasingly recognized that concentrated market power in local and global food supply chains has the potential to undermine the food systems transformations necessary to address current rates of NCDs [12, 13]. The International Panel of Experts on Sustainable Food Systems recently stated that “Dominant firms have become too big to feed humanity sustainably, too big to operate on equitable terms with other food system actors, and too big to drive the types of innovation we need’’ [14]. Indeed, several scholars have noted that the creation of healthy and sustainable food systems will require increased attention to complex challenges such as the highly integrated nature of a food system increasingly governed by multinational corporations and international investors, the rise in multi-stakeholder partnerships and market-oriented forms of governance, as well as the intensification of market concentration and power along food chains [14–16].

Corporate market power can be assessed through analyses of three interrelated concepts of market: 1) structure; 2) conduct; and 3) performance. The structure of an industry or market (e.g., the concentration of a market and degree of common shareholder ownership) can both influence and be influenced by the conduct of firms (i.e., firm behavior and strategy such as the pursuit of mergers and acquisitions), which can in turn influence or be influenced by industry or firm performance (e.g., profits and shareholder returns) [10]. Market structure analyses are an integral step in market power analyses and can provide important insight into the power held by leading corporations; however, there has been only limited analysis of market structure from a public health perspective to date. Most relevant studies pertaining to market structure have focused on supranational markets or analyses within the agricultural sector only [14, 16–19]. 

Research has demonstrated that Canadian food environments could be more conducive to healthy dietary patterns necessary for NCD prevention. For example, diets of higher nutritional quality that meet national dietary guidelines may be more expensive compared to less healthy counterparts [20, 21]. Moreover, the promotion of unhealthy food and beverages to children is widespread in the country [22] and industry compliance with national sodium reduction targets has been limited [23]. Better understanding structural factors that may facilitate and/or hinder positive changes to Canadian food environments is necessary.

This study aimed to descriptively analyze the structure of the Canadian food and beverage manufacturing and grocery retailing sectors as of 2020/21. The objectives were to identify the leading food and beverage manufacturing and grocery retailing companies in Canada, and understand the structure and competitive landscape of the markets in which these firms operate using a public health lens.

## Methods

This paper drew upon a theoretical framework developed to understand corporate market power from a public health perspective using the structure-conduct-performance model [10], and an applied market structure analysis which aimed to compare differences and similarities in market structure across European countries and potential implications for food environment policy [18]. The current study adapted the methods applied in Europe [18] for a national-level market structure analysis.

### Assessing the size of relevant markets

The most recent market size data (as off-trade/retail value retail selling price (RSP), which represents sales from retail settings excluding the sales tax) were obtained from Passport by Euromonitor International [24] for packaged food and non-alcoholic beverage manufacturing and modern grocery retailing (hereafter referred to as ‘grocery retailing’) sectors, including disaggregated data for 16 packaged food product markets (‘confectionery’, ‘ice cream and frozen desserts’, ‘savory snacks’, ‘sweet biscuits, snack bars and fruit snacks’, ‘ready meals’, ‘sauces, dressings and condiments’, ‘soups’, ‘sweet spreads’, ‘dairy’, ‘baked goods’, ‘breakfast cereals’, ‘processed fruits and vegetables’, ‘processed meat, seafood and alternatives’, ‘rice, pasta and noodles’, ‘edible oils’, and ‘baby foods’), 8 non-alcoholic beverage product markets (‘carbonates’, ‘fruit and vegetable juice’, ‘bottled water’, ‘concentrates’, ‘ready-to-drink (RTD) tea’, ‘RTD coffee’), and 5 types of grocery retailers (‘hypermarkets’, ‘supermarkets’, ‘discounters’, ‘forecourt retailers’, ‘convenience stores’) from 2012 to 2021 (all historical data available for download on Passport were included).

The relative size of each market was calculated in terms of the percent contribution of each market to their sector as of 2021:

$$\mathrm{Relative\ market\ size}= \frac{\mathrm{value\ of\ a\ market}}{\mathrm{value\ of\ a\ sector}}$$

For example, the relative size of the ‘carbonates’ market would be calculated as:

$$\mathrm{Relative\ size\ of\ 'carbonates'\ { market}}= \frac{\mathrm{Off}-\mathrm{trade\ value\ of\ 'carbonates'\ market}}{\mathrm{Off}-\mathrm{trade\ value\ of\ non}-\mathrm{alcoholic\ beverage\ sector}}$$

The change in relative market size since 2012 was calculated to assess changes over time as: relative market size in 2021 – relative market size in 2012.

As aggregated data were only available for the packaged food sector from 2016–2020, the retail value of the packaged food sector was calculated as the sum of the retail values of all (*n* = 16) packaged food product markets. Analyses were conducted for all markets described above with the exception of ‘edible oils’, and ‘baby foods’, the two smallest packaged food product markets, each accounting for less than 1% of sales within the packaged food sector.

### Identifying and describing relevant companies

The most recent national (i.e., ‘national brand owner’) and global (i.e., ‘global brand owner’) company share data were obtained from Passport by Euromonitor International [24] for the sectors and markets described above, including historical data going back 10 years (2012–2021). National brand owners (i.e., producers or distributors of a brand at the national level [25]) with ≥ 1% of shares within the packaged food manufacturing, non-alcoholic beverage manufacturing and/or grocery retailing sector(s) according to the most recent data (2020/21) were first identified as the largest contributors to Canadian sectors and markets. Company characteristics were collated from Refinitiv Eikon, a financial market database [26], and supplemented with targeted online searches on company websites, and/or information from MarketLine, a commercial intelligence database which profiles companies and markets [27]. Characteristics included company type (i.e., ‘public’ or ‘private’ company), headquarter location (i.e., ‘Canada’ or ‘foreign’ headquarters), as well as the name, type, and headquarter location of the associated parent company if applicable (e.g., if the national brand owner was a subsidiary of another company). Companies were also identified as being either ‘multinational’ companies, if they or their parent company had operations (e.g., production or retail facilities) in > 1 country, or ‘national’ companies.

Descriptive statistics examined the proportion of the market share held by public (vs private), multinational (vs national) and foreign multinational companies by sector.

### Measuring market concentration

Market concentration was then assessed. Market concentration, referring to the extent to which market shares are concentrated between firms active in the market in question, is often used as an indicator for the level of competition within a market, such that higher concentration suggests lower levels of competition within a market [28, 29]. The 4-firm concentration ratio (CR4) and the Herfindahl Hirschman Index (HHI) are commonly used market concentration metrics [30]. The CR4 is measured by adding the market shares of the top 4 firms in a market [31]. A value below 40 can be considered as being suggestive of a competitive market, whereas a value above 60 suggestive of a market dominated by either one or a few firms (referred to as a monopolistic or oligopolistic market) [30]. The HHI is calculated by summing the square of the market shares of all companies within a market [32], and therefore takes into account the distribution of market shares [31]. Various HHI thresholds have been developed to classify the degree of concentration in a market. According to the US Department of Justice’s Horizontal Merger Guidelines, an HHI value above 2500 would be suggestive of a highly concentrated market, an HHI between 1500 and 2500 a moderately concentrated market, and an HHI below 1500 an unconcentrated market [33]. The European Central Bank uses lower thresholds, considering values below 1000 to be indicative of unconcentrated markets, while those above 1800 to be indicative of highly concentrated markets [34]. Thresholds of 1000 and 2000 have also been used to assess the concentration of the European food and beverage market [18], values which were based on the European Union’s merger regulations (2004/C 31/03) [35]. In Canada, the Competition Bureau’s Merger Enforcement Guidelines do not establish HHI thresholds, however, a post-merger CR4 ≥ 65 is generally challenged in light of the potential threat to competition [36]. Market concentration is a more meaningful indicator when applied to a specific market of substitutable goods (e.g., ‘breakfast cereals’ rather than all ‘packaged foods’) within constrained geographical boundaries; market concentration levels may be underestimated when applied too broadly.

Market concentration was assessed for sectors and markets described above using both ‘national brand owner’ and ‘global brand owner’ market share data over 10 years (2012–2021). Metrics included: 1) the number of brand owners with ≥ 1% market share, 2) the 4-firm concentration ratio (CR4), and 3) the Herfindahl Hirschman Index (HHI) (using data for companies with ≥ 1% market share). Integrated thresholds based on aforementioned values were used to interpret HHI values, such that an HHI > 2500 was deemed suggestive of very high market concentration, 1800–2499 high concentration, 1500–1799 moderate concentration, 1000–1500 moderate-low concentration, and < 1000 low concentration.

### Assessing company ownership

Company shareholder ownership was subsequently assessed. Ownership data for all identified publicly listed companies were downloaded from Refinitiv Eikon in March 2022 [26]. If ownership data were not available for the national brand owner (i.e., if the national brand owner was privately owned by a publicly listed parent company), ownership data were downloaded for the identified publicly listed parent company. 

First, the top 3 investors (i.e., with the largest percent shareholder ownership) for each company were identified, including their name and percent shareholder ownership, and the total shares owned by the top 3 investors were assessed for each company, to capture the diversity of investors, and range in percent shareholder ownership held by top investors. Overall mean and median shares held by the top 3 investors were assessed.

Then, ownership by three of the largest global asset managers (i.e., Blackrock, The Vanguard Group Inc, and State Street Global Advisors [37, 38]) was assessed. Percent shareholder ownership by Blackrock Institutional Trust Company (a subsidiary of Blackrock), The Vanguard Group Inc and State Street Global Advisors (US) was extracted if the asset manager owned ≥ 1% of the public company’s shares.

The proportion of all publicly listed companies in which each of the three aforementioned asset manager owned ≥ 1% was calculated. Ownership networks were also mapped for each sector, using a scheme adapted from research conducted within the US seed industry [39], including shareholder ownership by Blackrock Institutional Trust Company, The Vanguard Group Inc and State Street Global Advisors (US).

## Results

### Size of relevant markets

Relative market size is presented in Table 1. As of 2021, the largest packaged food market was the 'dairy' product market, followed by the 'baked goods', 'processed meat, seafood and alternatives' and 'ready meals' product markets. The largest non-alcoholic beverage market was the 'fruit and vegetable juice' product market, followed by the 'carbonates' and 'bottled water' product markets. Within the grocery retailing sector, 'supermarkets', followed by 'hypermarkets' and 'discounters' constituted the largest markets.

**Table 1:** Size of packaged food and non-alcoholic beverage manufacturing and grocery retailing markets relative to their sector

	**Market**	**Relative size of market as of 2021** **(change since 2012) (%)**
**Packaged food manufacturing**^**a**^	Dairy	23 (-1)
	Baked goods	13 (0)
	Processed meats, seafood and alternatives	12 (1)
	Ready meals	11 (1)
	Savory snacks	8 (1)
	Confectionery	7 (0)
	Sauces, dressings and condiments	6 (0)
	Sweet biscuits, snack bars and fruit snacks	4 (0)
	Processed fruits and vegetables	4 (0)
	Rice, pasta and noodles	3 (0)
	Ice cream and frozen desserts	3 (0)
	Breakfast cereals	2 (-1)
	Sweet spreads	1 (0)
	Soups	1 (0)
**Non-alcoholic beverage manufacturing**	Fruit and vegetable juice	32 (-6)
	Carbonates	23 (-5)
	Bottled water	22 (3)
	RTD tea	9 (4)
	Energy drinks	7 (2)
	Sports drinks	5 (0)
	Concentrates	2 (0)
	RTD coffee	1 (1)
**Grocery retailing**	Supermarkets	43 (-5)
	Hypermarkets	29 (5)
	Discounters	23 (0)
	Forecourt retailers	4 (0)
	Convenience	1 (-1)

^a^The ‘Edible oils’ and ‘Baby foods’ packaged food product markets were excluded from analyses

### Leading food and beverage companies

A total of 34 national brand owners were identified as having ≥ 1% of shares in the packaged food manufacturing (*n* = 19), non-alcoholic beverage manufacturing (*n* = 13), and/or grocery retailing (*n* = 6) sector(s) (see Table 2). Of these, 2 firms (Loblaw and Sobeys) held ≥ 1% of shares in all 3 sectors. National brand owners included in this analysis accounted for 49% of shares within the packaged food manufacturing sector, 75% of shares within the non-alcoholic beverage manufacturing sector, and 86% of shares within the grocery retailing sector.

**Table 2:** Ownership structure of packaged food and non-alcoholic beverage manufacturers and grocery retailers

National brand owner with ≥ 1% market share in Canada	Sector (share [%])^a^	Parent company	Ownership status	Headquarter location	Company type
A. Lassonde Inc	B (5.7)	Lassonde Industries Inc	Public	Canada	Multinational
Sun-Rype Products Ltd	B (1.9)				
Agropur Cooperative	PF (3.8)	-	Private	Canada	Multinational
Alimentation Couche-Tard Inc	R (1.8)	-	Public	Canada	Multinational
BlueTriton Brands Inc^b^	B (7.3)	-	Private	Foreign	Multinational
Cadbury Adams Canada Inc^c^	PF (1.2)	Mondelez International Inc	Public	Foreign	Multinational
Mondelez Canada Inc	PF (1.1)				
Campbell Company of Canada^d^	B (1.0)	Campbell Soup Co	Public	Foreign	Multinational
Canada Bread Co (Bimbo Canada)	PF (1.4)	Grupo Bimbo SAB de CV	Public	Foreign	Multinational
Canada Dry Motts Inc	B (5.5)	Keurig Dr Pepper Inc	Public	Foreign	Multinational
Snapple Beverage Corp	B (1.2)				
Catelli Foods Corp^e^	PF (1.0)	Barilla Group	Private	Foreign	Multinational
Coca-Cola Ltd	B (13.4)	Coca Cola Co	Public	Foreign	Multinational
Minute Maid Co of Canada	B (7.9)				
Danone Canada Inc	PF (1.5)	Danone SA	Public	Foreign	Multinational
Frito-Lay Canada^f^	PF (3.4)	PepsiCo Inc	Public	Foreign	Multinational
PepsiCo Beverages Canada^f^	B (21.1)				
General Mills Canada Corp	PF (2.4)	General Mills Inc	Public	Foreign	Multinational
George Weston Ltd	PF (1.5)	-	Public	Canada	Multinational
Kellogg Canada Inc	PF (1.5)	Kellogg Co	Public	Foreign	Multinational
Kraft Heinz Canada ULC	PF (4.4)	Kraft Heinz Co	Public	Foreign	Multinational
Lactalis Canada Inc	PF (3.7)	Groupe Lactalis	Private	Foreign	Multinational
Loblaw Cos Ltd^g^	PF (6.1), B (4.6), R (24.6)	George Weston^f^	Public	Canada	National
Maple Leaf Foods Inc	PF (1.6)	Maple Leaf Foods Inc	Public	Canada	Multinational
Schneider Corp^h^	PF (1.8)				
Metro Inc	R (12.2)	-	Public	Canada	National
Nestlé Canada Inc	PF (3.8)	Nestlé SA	Public	Foreign	Multinational
Ocean Spray Cranberries Inc	B (1.4)	-	Private	Foreign	Multinational
Overwaitea Food Group	R (2.8)	Jim Pattison Group Inc	Private	Canada	Multinational
Red Bull Canada Ltd	B (2.5)	Red Bull GmbH	Private	Foreign	Multinational
Saputo Inc	PF (5.1)	-	Public	Canada	Multinational
Sobeys Inc	PF (2.1), B (1.4), R (24.1)	Empire Co Ltd	Public	Canada	National
Unilever Canada Inc	PF (1.2)	Unilever PLC	Public	Foreign	Multinational
Wal-Mart Canada Inc	R (20.6)	Walmart Inc	Public	Foreign	Multinational

^a^Source: © Euromonitor International [24]. *Abbreviations*: *B* Non-alcoholic beverage, *PF* Packaged Food, *R* Grocery retail

^b^Nestlé Waters North America (formerly owned by Nestlé SA) changed its name to BlueTriton Brands after its acquisition by One Rock Capital Partners, LLC and Metropoulos & Co in March 2021 [40]

^c^Data for Mondelez Canada Inc and Cadbury Adams Canada are reported separately on Passport. Cadbury was acquired by Kraft Foods in 2010, and the plant became part of the Mondelez International group in 2012 [41]

^d^Data is reported for Campbell Soup Co on Passport; The Campbell Company of Canada is the Canadian subsidiary of Campbell Soup Co

^e^In 2021, Barilla acquired the Catelli dry pasta business, including the Catelli, Lancia, and Splendor brands and the production facilities in Montreal, Quebec [42]

^f^PepsiCo Canada is composed of two business units: PepsiCo Beverages Canada and PepsiCo Foods Canada (which includes Frito Lay Canada) [43]

^g^Loblaw Cos Ltd is a publicly owned Canadian company; information about the company is provided independently of its affiliation with George Weston Ltd. Nonetheless, Loblaw Cos Ltd is an operating segment of George Weston Ltd [44]

^h^Schneider Corp was acquired by Maple Leaf Foods Inc in 2004 [45]. Data for Schneider and Maple Leaf are reported separately on Passport

A total of 29 parent companies accounted for all national brand owners included in this analysis. Most parent companies were publicly listed; 83% of sampled shares within the packaged food manufacturing sector, 85% of sampled shares within the non-alcoholic beverage manufacturing sector, and 97% of the sampled shares within the grocery retailing sector were accounted for by national brand owners which were or were owned by publicly listed parent companies. Multinational companies accounted for 83% of the sampled shares within the packaged food manufacturing sector, 92% of the shares within the non-alcoholic beverage manufacturing sector, and 29% of the shares within the grocery retailing sector. Foreign multinational companies accounted for 55% of the sampled shares held by packaged food manufacturers, 82% of the shares held by non-alcoholic beverage manufacturers, and 24% of the shares held by grocery retailers included in this analysis.

### Market concentration

Table 3 summarizes the level of concentration (CR4 and HHI) of Canadian packaged food and non-alcoholic beverage manufacturing and grocery retailing markets using national brand owner data as of 2021. Market concentration metrics over 10 years stemming from both national brand owner and global brand owner data (to support international comparisons) are presented in Supplementary Tables A1, A2 and A3.

**Table 3:** Concentration of Canadian packaged food and non-alcoholic beverage manufacturing and grocery retailing markets as of 2021, and change in value since 2012

	**Market**	**Four-firm concentration ratio as of 2021** **(change since 2012)**	**Herfindahl–Hirschman Index as of 2021** **(change since 2012)**
**Packaged food manufacturing**	Soups	81 (-2)	3700 (-104)
	Ice cream and frozen desserts	77 (-2)	2041 (-409)
	Breakfast cereals	77 (0)	2005 (-163)
	Dairy	63 (-1)	1205 (2)
	Savory snacks	52 (-1)	1467 (-66)
	Rice, pasta and noodles	52 (7)	1017 (286)
	Sweet spreads	52 (1)	869 (21)
	Processed fruits and vegetables	50 (5)	721 (119)
	Sauces, dressings and condiments	49 (6)	995 (373)
	Confectionery	48 (1)	719 (47)
	Baked goods	42 (-1)	473 (-15)
	Processed meats, seafood and alternatives	40 (-3)	511 (-69)
	Sweet biscuits, snack bars and fruit snacks	38 (-6)	463 (-151)
	Ready meals	34 (-2)	458 (-48)
**Non-alcoholic beverage manufacturing**	Sports drinks	99 (1)^a^	6483 (-88)
	Energy drinks	84 (3)	2440 (-79)
	Carbonates	80 (1)	2067 (-117)
	Concentrates	74 (-6)	2262 (-380)
	RTD tea	70 (-8)	1923 (-632)
	RTD coffee	62 (-20)^a^	1462 (-5279)
	Bottled water	62 (-3)	1364 (-137)
	Fruit and vegetable juice	57 (4)	999 (112)
**Grocery retailing**	Hypermarkets	100 (0)^b^	5959 (747)
	Supermarkets	87 (1)	3219 (1018)
	Discounters	84 (5)	2405 (-249)
	Forecourt retailers	62 (5)	1728 (286)
	Convenience	53 (1)^a^	1576 (304)

^a^Interpret with caution; data available for < 4 companies 1 or more years between 2012 and 2021

^b^ < 4 companies held 100% of the market

#### Market concentration within packaged food and non-alcoholic beverage manufacturing sectors 

As of 2021, CR4 values ranged from 34 to 81 across packaged food product markets (median CR4 = 51), while HHI values ranged from 458 to 3700 (median HHI = 932). CR4 values were < 40% (low concentration) for 2 of 14, 40–60% for 8 of 14 (moderate concentration) and > 60% (high concentration) for 4 of 14 packaged food product markets. Overall, 8 of 14 packaged food product markets had an HHI < 1000 (low concentration) and 3 of 14 1000- < 1500 (low-moderate concentration), while 2 of 14 had an HHI > 1800–2500 (high concentration) and 1 of 14 > 2500 (very high concentration). Substantial fluctuations in HHI values over the past 10 years were observed for certain packaged food product markets, particularly for the ‘sauces, dressings and condiments’ (+ 373), ‘ice cream and frozen desserts’ (-409), and ‘rice, pasta and noodles’ (+ 286) product markets.

As of 2021, CR4 values ranged from 57 to 99 across non-alcoholic beverage product markets (median CR4 = 72), while HHI values ranged from 999 to 6483 (median HHI = 1995). CR4 values were > 60% for all non-alcoholic beverage product markets except for the ‘fruit and vegetable juice’ market. Overall, 1 of 8 product markets resulted in an HHI < 1000, 2 of 8 1000- < 1500, 4 of 8 > 1800–2500, and 1 of 8 > 2500. The largest fluctuations in HHI values over the past 10 years were observed for the ‘RTD coffee’ (-5279), followed by the ‘RTD tea’ (-632) product markets.

#### Market concentration within the grocery retailing sector

As of 2021, CR4 values ranged from 53 to 100 across grocery retailing markets (median CR4 = 84), while HHI values ranged from 1576 to 5959 (median HHI = 2405). CR4 values for and within the Canadian grocery retailing sector were all ≥ 60%, while HHI values were all > 1500, with 1 of 5 > 1800–2500 (i.e., ‘discounters’), and 2 of 5 > 2500 (i.e., ‘hypermarkets’, ‘supermarkets’). A substantial rise in HHI values was observed within the grocery retailing sector between 2012 and 2013, specifically for ‘supermarkets’ (see Supplementary Table A3).

### Company ownership

#### Shareholder ownership of publicly listed companies by any investor

The percent shareholder ownership of each publicly listed national brand owner (or their publicly listed parent company) by investors varied substantially (see Table 4); the percent share owned by the top investor varied between 4.09% (i.e., BMO Asset Management Inc’s shareholder ownership of Sobeys) and 53.56% (i.e., Willard Galen Garfield’s shareholder ownership of George Weston Ltd). Total shares of companies owned by the top 3 investors ranged from 10.56% to 58.69% of total shares. Median shareholder ownership by the top 3 investors (for each publicly listed company) totaled 26.43%, while the mean totaled 31.76%.

**Table 4:** Shareholder ownership of publicly listed packaged food manufacturers, non-alcoholic beverage manufacturers and grocery retailers in Canada

National brand owner with ≥ 1% market share in Canada	Parent company	Top 3 investors (percent ownership [%]) ^a^	Percent ownership by 3 large asset managers (%) ^b^		
			The Vanguard Group, Inc	BlackRock Institutional Trust Company	State Street Global Advisors (US)
A. Lassonde Inc	Lassonde Industries Inc	QV Investors Inc. (14.54) Fidelity Management & Research Company LLC (7.30) Tweedy, Browne Company LLC (4.59)	-	-	-
Sun-Rype Products Ltd					
Alimentation Couche-Tard Inc	-	Développements Orano, Inc. (9.97) D'Amours (Jacques) (5.7) Fortin (Richard) (3.13)	2.01	1.17	-
Cadbury Adams Canada Inc	Mondelez International Inc	The Vanguard Group, Inc. (8.18) State Street Global Advisors (US) (4.61) BlackRock Institutional Trust Company, N.A. (4.31)	8.18	4.31	4.61
Mondelez Canada Inc					
Campbell Company of Canada	Campbell Soup Co	Malone (Mary Alice D) (17.66) Dorrance (Bennett) (14.89) The Vanguard Group, Inc. (7.25)	7.25	3.89	3.46
Canada Bread Co (Bimbo Canada)	Grupo Bimbo SAB de CV	Normaciel, S.A. de C.V. (39.25) Promociones Monser, S.A. de C.V. (12.3) Philae, S.A. de C.V. (4.95)	1.58	-	-
Canada Dry Motts Inc	Keurig Dr Pepper Inc	Maple Holdings BV (32.88) Mondelez International Inc (5.33) BDT Capital Partners, LLC (4.82)	3.19	1.53	1.09
Snapple Beverage Corp					
Coca-Cola Ltd	Coca Cola Co	Berkshire Hathaway Inc. (9.23) The Vanguard Group, Inc. (7.89) BlackRock Institutional Trust Company, N.A. (4.12)	7.89	4.12	3.93
Minute Maid Co of Canada					
Danone Canada Inc	Danone SA	BlackRock Institutional Trust Company, N.A. (6.15) MFS Investment Management (4.99) Artisan Partners Limited Partnership [Activist] (4.95)	2.40	6.15	-
Frito-Lay Canada	PepsiCo Inc	The Vanguard Group, Inc. (8.86) BlackRock Institutional Trust Company, N.A. (4.73) State Street Global Advisors (US) (4.26)	8.86	4.73	4.26
PepsiCo Beverages Canada					
General Mills Canada Corp	General Mills Inc	The Vanguard Group, Inc. (8.41) State Street Global Advisors (US) (5.71) Capital International Investors (5.61)	8.41	5.10	5.71
George Weston Ltd	-	Weston (Willard Galen Garfield) (53.56) RBC Global Asset Management Inc. (3.68) CIBC Asset Management Inc. (1.45)	1.33	-	-
Kellogg Canada Inc	Kellogg Co	Kellogg W.K. Foundation Trust (17.12) The Vanguard Group, Inc. (8.40) Gund (Gordon) (6.39)	8.40	5.19	4.19
Kraft Heinz Canada ULC	Kraft Heinz Co	Berkshire Hathaway Inc. (26.61) 3G Capital Management, Inc. (15.14) The Vanguard Group, Inc. (4.57)	4.57	2.58	2.61
Loblaw Cos Ltd	*George Weston*	George Weston Ltd (47.21) RBC Global Asset Management Inc. (1.60) TD Asset Management Inc. (1.41)	1.29	-	-
Maple Leaf Foods Inc	Maple Leaf Foods Inc	McCain (Michael Harrison) (39.04) RBC Global Asset Management Inc. (8.60) The Vanguard Group, Inc. (1.61)	1.61	-	-
Schneider Corp					
Metro Inc	-	Fidelity Management & Research Company LLC (17.26) TD Asset Management Inc. (2.97) The Vanguard Group, Inc. (2.76)	2.76	1.74	-
Nestlé Canada Inc	Nestlé SA	BlackRock Institutional Trust Company, N.A. (5.04) Capital Research Global Investors (3.81) The Vanguard Group, Inc. (2.72)	2.72	5.04	-
Saputo Inc	-	Jolina Capital, Inc. (31.40) Placements Italcan Inc (10.24) Beutel, Goodman & Company Ltd. (2.32)	1.53	-	-
Sobeys Inc	Empire Co Ltd	BMO Asset Management Inc. (4.09) CI Global Asset Management (3.53) Jarislowsky Fraser, Ltd. (2.94)	2.65	1.22	-
Unilever Canada Inc	Unilever PLC	BlackRock Institutional Trust Company, N.A. (6.57) The Vanguard Group, Inc. (3.17) Leverhulme Trust (1.83)	3.17	6.57	-
Wal-Mart Canada Inc	Walmart Inc	Walton Enterprises, L.L.C. (46.59) The Vanguard Group, Inc. (4.51) BlackRock Institutional Trust Company, N.A. (2.25)	4.51	2.25	2.19

^a^Data obtained from Refinitv Eikon in March 2022

^b^Percent shareholder ownership if ≥ 1.00% of outstanding shares in the publicly listed company

#### Shareholder ownership of publicly listed companies by three major asset managers

**Fig. 1:**
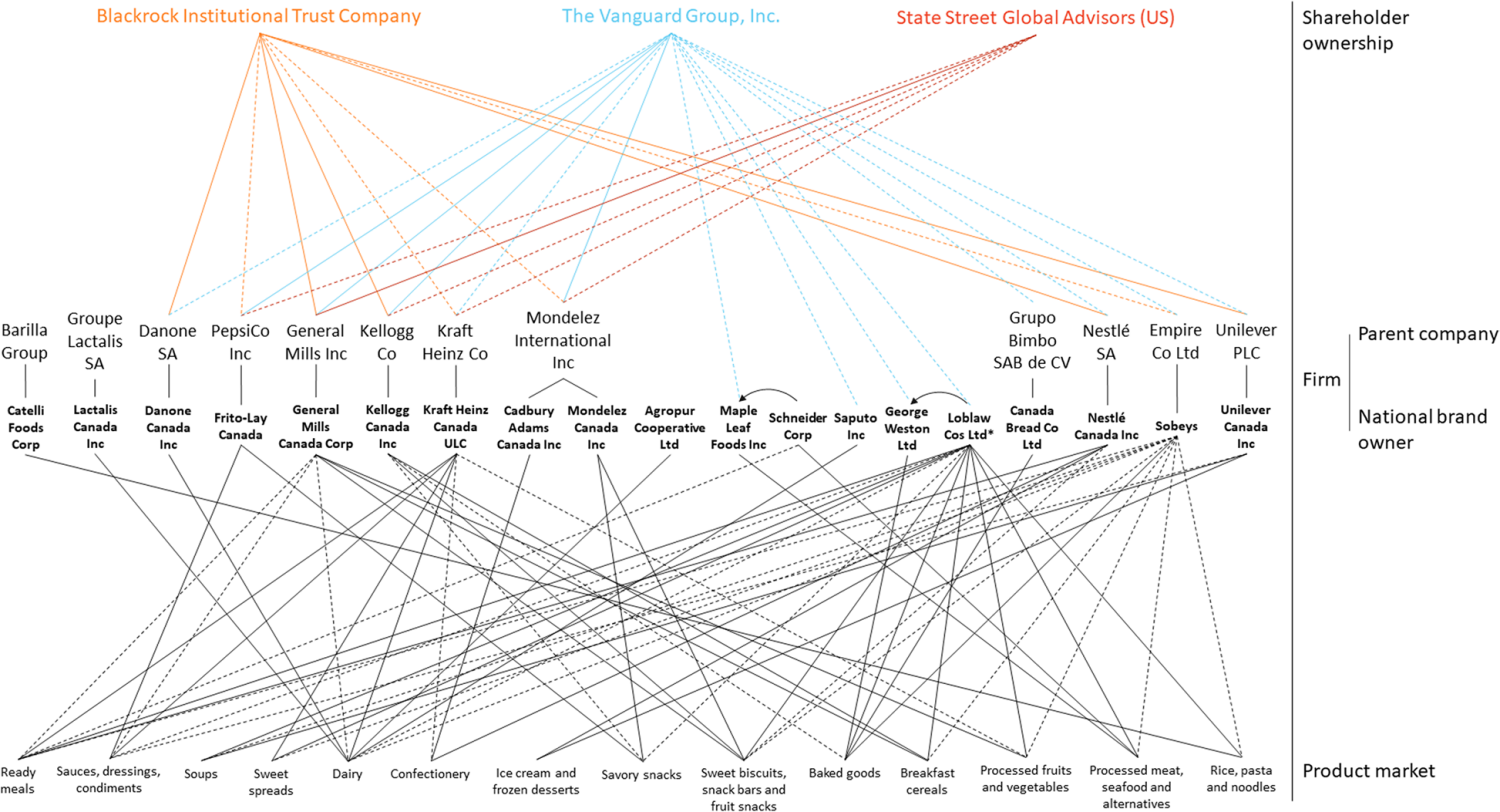
Ownership networks within the Canadian packaged food manufacturing sector; Figure 1 maps ownership within the Canadian packaged food manufacturing sector including ownership by the Vanguard Group Inc, Blackrock Institutional Trust Company and State Street Global Advisors (US). **A** Shareholder ownership level: Dotted lines represent ≥ 1% ownership while solid lines represent ≥ 5% ownership by three major institutional investors; **B** Firm level: All national brand owners with ≥ 1% of shares within the Canadian packaged food sector are represented. National brand owners are associated with their parent company (if applicable). *Loblaw Cos Ltd is a publicly listed company, however, George Weston Ltd is its parent company. **C** Product market level: All national brand owners are linked to a specific product market if they account for ≥ 1% of shares within the product market

**Fig. 2:**
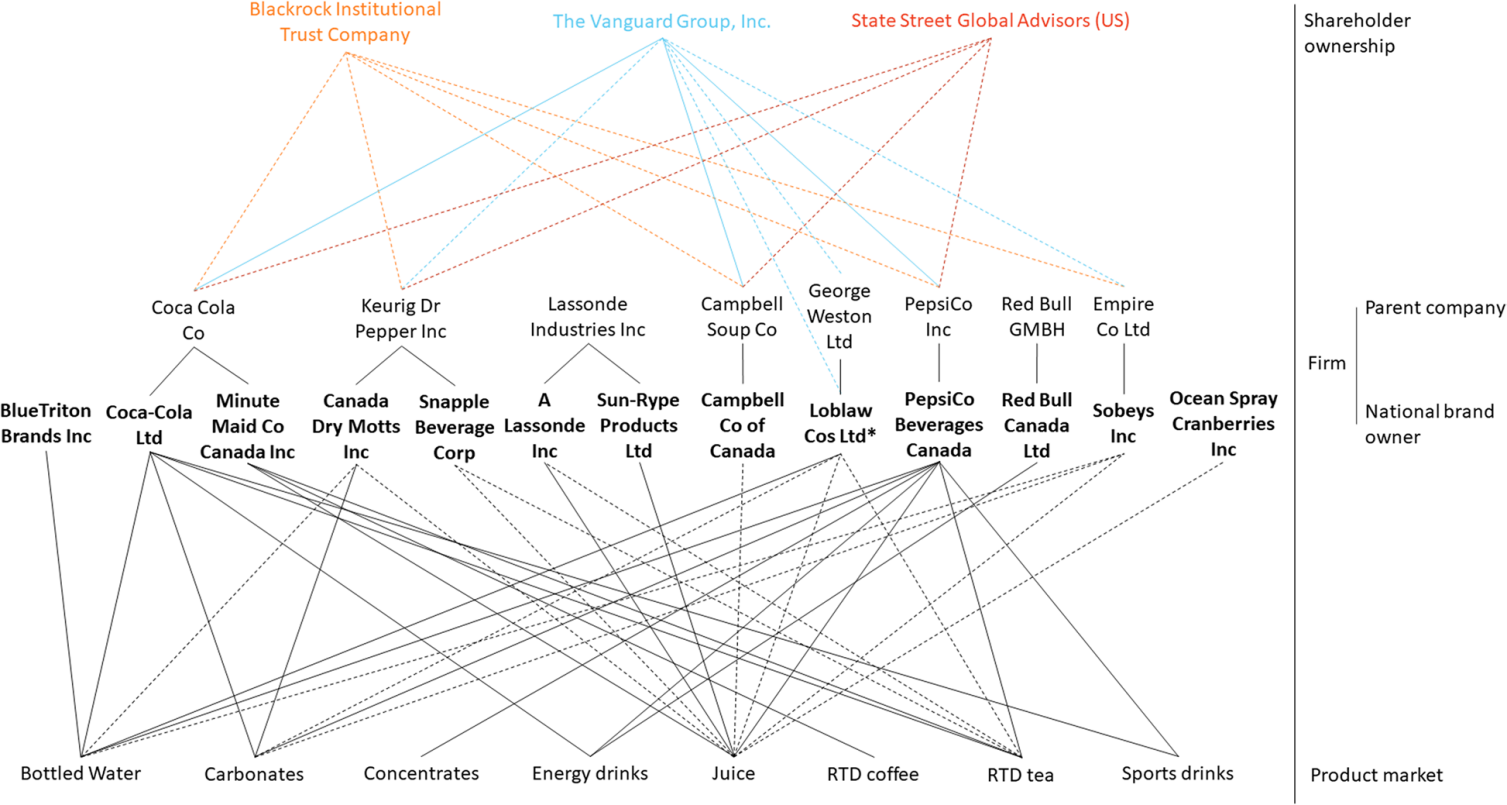
Ownership networks within the Canadian non-alcoholic beverage manufacturing sector; Figure 2 maps ownership within the Canadian non-alcoholic beverage manufacturing sector including ownership by the Vanguard Group Inc, Blackrock Institutional Trust Company and State Street Global Advisors (US). **A** Shareholder ownership level: Dotted lines represent ≥ 1% ownership while solid lines represent ≥ 5% ownership by three major institutional investors; **B** Firm level: All national brand owners with ≥ 1% of shares within the Canadian non-alcoholic beverage sector are represented. National brand owners are associated with their parent company (if applicable). *Loblaw Cos Ltd is a publicly listed company, however, George Weston Ltd is its parent company. **C** Product market level: All national brand owners are linked to a specific product market if they account for ≥ 1% of shares within the product market

**Fig. 3:**
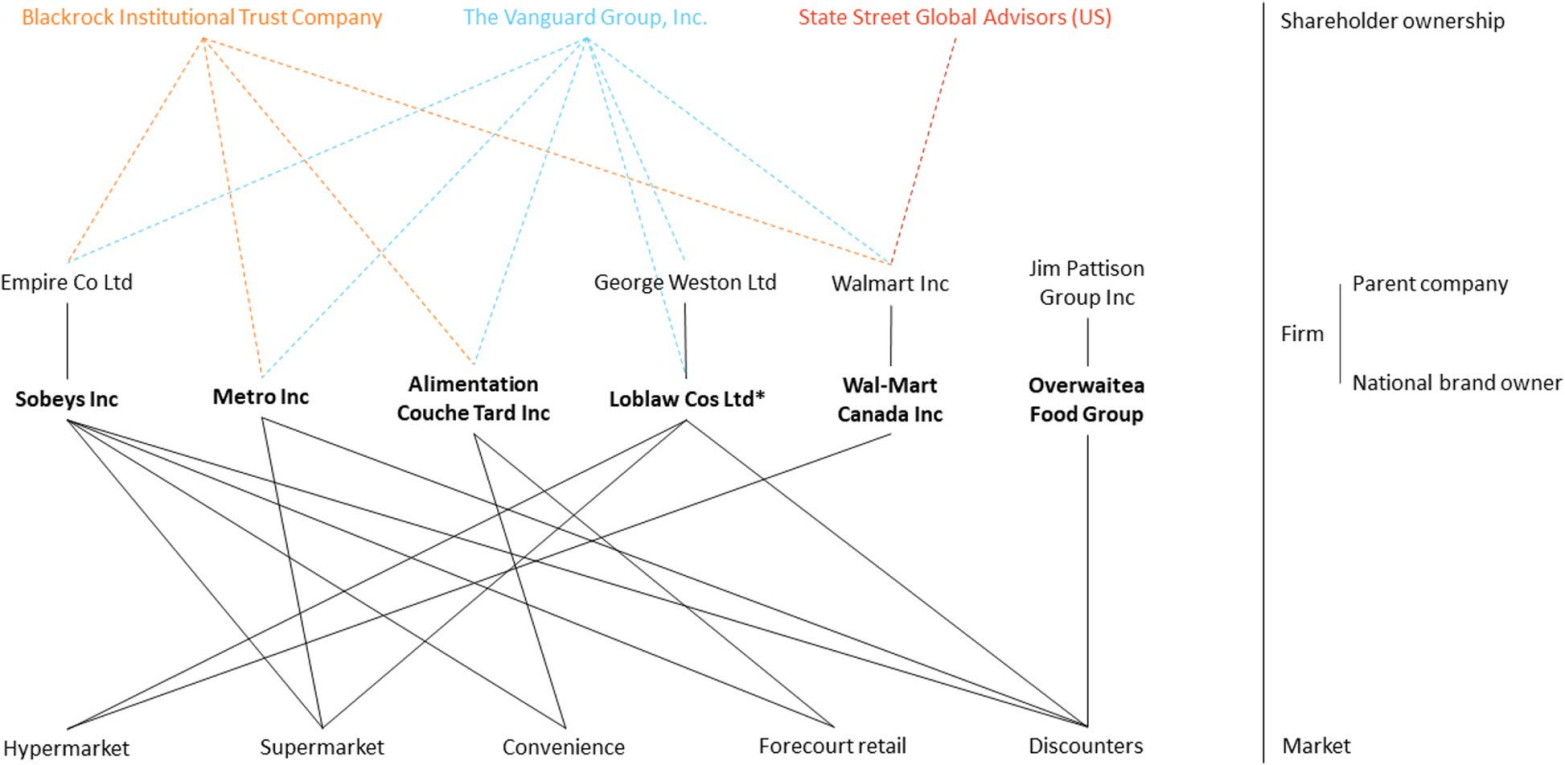
Ownership networks within the Canadian grocery retailing sector; Figure 3 maps ownership within the Canadian grocery retailing sector including ownership by the Vanguard Group Inc, Blackrock Institutional Trust Company and State Street Global Advisors (US). **A** Shareholder ownership level: Dotted lines represent ≥ 1% ownership while solid lines represent ≥ 5% ownership by three major institutional investors; **B** Firm level: All national brand owners with ≥ 1% of shares within the Canadian grocery retailing sector are represented. National brand owners are associated with their parent company (if applicable). *Loblaw Cos Ltd is a publicly listed company, however, George Weston Ltd is its parent company. **C** Market level: All national brand owners are linked to a specific disaggregated market if they account for ≥ 1% of shares within that market

Figures 1, 2 and 3 map networks of ownership by the Vanguard Group Inc, Blackrock Institutional Trust Company and State Street Global Advisors (US) within Canadian food and beverage sectors and markets. Overall, The Vanguard Group Inc, Blackrock Institutional Trust Company and State Street Global Advisors (US) owned shares in the majority of national brand owners (or their parent company), including many that operate within the same sectors and markets (see Table 4 and Figs. 1, 2 and 3). The Vanguard Group Inc owned ≥ 1% of shares in 95% of publicly listed companies, Blackrock Institutional Trust Company 71%, and State Street Global Advisors (US) 43%.

## Discussion

Overall, the Canadian packaged food and non-alcoholic beverage manufacturing sectors consisted of both oligopolistic and more competitive product markets with significant foreign multinational company presence, in contrast with the grocery retailing sector which was highly concentrated and dominated by national companies. There was considerable evidence of common ownership within and across all sectors.

### Market concentration

The Canadian packaged food sector was comprised of product markets of various sizes, and with varying levels of concentration. Analyses within specific product markets revealed some moderately concentrated markets (CR4 > 40 and HHI > 1000) (i.e., ‘dairy’, ‘savory snacks’, and ‘rice, pasta and noodles’), and some highly concentrated (CR4 > 60 and HHI > 1800) markets (i.e., ‘soups’, ‘ice cream and frozen desserts’ and ‘breakfast cereals’), consistent with a recent analysis of the European single market, where ‘soups’, ‘ice cream and frozen desserts’, and ‘breakfast cereals’ were found to be the most concentrated packaged food product markets [18]. Within the non-alcoholic beverage sector, many product markets (i.e., ‘carbonates’, ‘concentrates’, ‘energy drinks’, ‘RTD tea’ and ‘sports drinks’) were highly concentrated which was similarly seen in Europe within the ‘carbonates’, ‘energy drinks’, ‘sports drinks’ and ‘RTD tea’ product markets, and distinctively within the ‘RTD coffee’ product market [18]. Overall, these findings suggest important (although perhaps unsurprising) similarities in the structure of Western food and beverage manufacturing markets.

Minor variations and fluctuations in market concentration metrics were noted over the past ten years within the packaged food sector. Certain changes can be explained by horizontal mergers and acquisitions. For instance, in 2015, an increase in the HHI value (from 595 to 938) for the ‘sauces, dressings and condiments’ product market was seen, likely as a result of the merger between the Heinz Company of Canada Ltd, and Kraft Canada. In the non-alcoholic beverage sector, a steep decrease was seen in the HHI values for the ‘RTD coffee’ product market over time. Distinctively, this drop in concentration may be explained by the emergence of companies offering products within this market; as of 2012, a single company held over 80% of the shares in the market, which decreased substantially over the following 2 years, in tandem with rapid market growth [24]. 

In contrast with the packaged food and non-alcoholic beverage sectors, the grocery retailing sector was dominated by national companies (with the exception of Wal-Mart). Four competing firms (i.e., Sobeys Inc, Metro Inc, Loblaw Co Ltd, and Wal-Mart Canada Inc) largely dominated the sector (median CR4 = 84%). Although not amongst the top 4 companies within the grocery retailing sector, Alimentation Couche Tard Inc was a leading company within the ‘Convenience’ and ‘Forecourt retail’ markets, and the geographic access to its outlets was greatest among all leading grocery retailers in 2021 [46]. While providing a substantially smaller volume of sales, the convenience and forecourt retailing sectors are of relevance to public health given they may have a product selection of poorer nutritional quality, compared to that of larger retailers such as supermarkets [47].

Taken together, these data suggest high market concentration and a lack of competition in the grocery retailing sector in Canada, as has been identified in other countries [18, 48]. These structural characteristics of the Canadian retail sector likely indicates that a small set of companies have extensive market power in this sector. For example, due to the oligopolistic nature of the markets in which they operate, retailers may have substantial buyer power over suppliers (e.g., manufacturers), and seller power over consumers [49]. This market power is likely exacerbated because many of the retailers are also highly vertically integrated and produce and sell their own brands. Excessive retailer market power may have important public health implications as retailers are gatekeepers of modern food systems [48]. In line with this finding, the Canadian Competition Bureau has recently launched an investigation into competition within the grocery retailing sector in light of rising food prices within the country [50]. 

Strong nutrition-related policies and action amongst the small number of leading Canadian grocery retail companies, including, to a lesser extent, convenience and forecourt retailers, would likely have an impact on a large number of consumers given that 73% of Canadian food expenditures are spent in retail settings [51]. Opportunities to improve healthfulness within retailers could include policies that address the promotion of ‘less healthy’ foods, the availability of and access to ‘healthier’ and ‘less healthy’ foods (e.g., at check-out points), as well as the nutrition information provided in stores (e.g., for ready-to-eat foods and own-brand products) and online [52]. Implementation of such policies could be conducive to making healthier choices easier for consumers in environments where they make the majority of their food purchases. Similarly, strong nutrition-related action, such as addressing nutrients of concern in leading products, by both retailers that produce and/or distribute own-brand products and leading manufacturers in product markets that are concentrated and/or generate important sales revenues, could potentially have significant public health implications.

### Company ownership

Ownership of packaged food and non-alcoholic beverage manufacturing and grocery retailing sectors was found to be highly complex and integrated. Most national brand owners, particularly within manufacturing sectors, were affiliated to a parent company, most often a foreign multinational.

In addition, many publicly listed companies had common investors as demonstrated by shareholder ownership by three large asset managers. Although more research is needed to fully understand the effect of common ownership on the level of competition, concerns exist over the potential for common ownership to reduce competition, particularly in concentrated markets [53]. The issue of common ownership from a public health perspective requires additional consideration, particularly in the concentrated grocery retailing sector, and in highly concentrated food and beverage product markets.

### Policy implications and areas for future investigation

These analyses underscore the globalized nature of modern Canadian food and beverage sectors, and the need for targeted and meaningful international efforts from the food industry to make positive changes that will support health, as called for by the World Health Organization and others [54, 55]. From a regulatory standpoint, while public health-related efforts from individual countries may help support changes within borders, cohesive and aligned policies across multiple countries are likely to have a greater impact, particularly when it comes to the packaged food and non-alcoholic beverage manufacturing sectors.

Research from various countries has shown different levels of commitments from food and beverage companies to support the transition towards healthier food environments [55–58]. However, research also suggests that voluntary company commitments to date have not necessarily translated into meaningful improvements or action in relation to marketing or the nutritional quality of the food supply (e.g., companies reporting stronger commitments regarding product (re)formulation have not further improved the healthfulness of their product portfolios compared to those with weaker commitments in Canada) [59–62]. Further investigation into the nutrition-related policies and actions of companies identified in this analysis is warranted, to increase the transparency and accountability of the private sector for their role in NCD prevention, identify areas for improvement, and/or draw attention to the need for further public sector policy action, as warranted [63].

Previous research has also identified that investors have significant potential to contribute to addressing nutrition-related challenges and increasing the accountability of food and beverage companies [3, 64–66]. For instance, following pressure from shareholders, Unilever recently committed to publicly reporting the healthfulness of its food sales using government-endorsed Nutrient Profile Models as well as internal metrics [67]. Although nutrition is only recently emerging as a potential focus area for responsible investment, it has been posited that investors would likely benefit from companies taking into account “nutrition-related risks and opportunities” such as the increasing demand for healthier products, the implementation of regulations pertaining to food composition, fiscal policies (e.g., taxes on sugary drinks) and the demand for product innovation, as these could influence their financial performance [64]. Nutrition-related considerations are garnering interest from institutional investors, however, targeted actions on these issues by institutional investors is still infrequent and inconsistent [3].

This study used a public health lens to better understand elements of market structure that may influence the healthfulness of food environments in Canada building on monitoring and accountability efforts as part of the International Network for Food and Obesity/non-communicable disease Research, Monitoring and Action Support (INFORMAS) [68]. Future market structure analyses may consider a multiple lens approach which incorporates health, environmental sustainability, equity and social justice, to further assess the suitability and effectiveness of current market regulations, and garner support for change where needed.

### Strengths and limitations

This study is the first investigation of food and beverage market structure from a public health perspective in Canada. It used a wide variety of indicators to assess market structure, including market size, number of active brand owners with a market share of ≥ 1%, level of market concentration, and company ownership. Nonetheless, further analyses of market structure could account for additional metrics such as the degree of vertical integration, barriers to market entry [10] and market dynamicity (i.e., the entry of new products within markets). For instance, certain manufacturers have operations upstream or downstream along the food chain, such as the Kraft Heinz company that not only produces packaged foods, but provides almost a third of processing tomato seeds across the globe [69]. Other companies are cooperatives and inherently operate along multiple segments along the value chain. For example, Agropur Cooperative is owned by 2908 dairy producers whose milk is used to produce a variety of dairy products [70]. As such, the Canadian food system is even more integrated than this analysis would suggest. Moreover, company and brand ownership are dynamic, with companies frequently and strategically selling or acquiring brands or companies, and monitoring the structure of leading companies can help understand how they maintain or gain market power.

Several of the analytical variables used in this paper have limitations. HHI values likely present an underestimation of the level of concentration within the Canadian packaged food and non-alcoholic beverage manufacturing and grocery retailing markets, for several reasons. First, certain product markets which were assessed constituted of multiple smaller product markets (e.g., the ‘dairy’ product market included products such as butter, drinkable yogurt and cheese). Next, national geographical boundaries were used to assess market concentration, however, while not available via Passport, by Euromonitor International, smaller geographical boundaries may have been relevant to define grocery retailing markets; for instance, Metro Inc was identified as a leading grocery retailer in Canada, yet only operates in Eastern Canada (i.e., in the provinces of Québec and Ontario). Lastly, metrics were assessed for brand owners with ≥ 1% market share (as opposed to using data for those with even the smallest % market share), as information for all brand owners active within a market is not always available on Passport.

Finally, this analysis focused on market structure, however, structure, conduct and performance are inter-related concepts of corporate market power. Future work could examine the conduct of the identified companies (and investors) and their performance focusing on public health and sustainability outcomes, perhaps using or adapting INFORMAS protocols [68].

## Conclusion

An analysis of the Canadian food and beverage market demonstrated the globalized and integrated nature of the Canadian packaged food and non-alcoholic beverage manufacturing and grocery retailing sectors. Moderate to high levels of market concentration in numerous product markets and in the grocery retailing sector suggest that efforts by leading companies could significantly benefit the healthfulness of those product markets, and retail settings in which Canadians make the majority of their food selections. Better understanding market structure may help identify additional levers to improve the healthfulness of the Canadian food environment, and has the potential to guide further in-depth analyses pertaining to industry policies and practices related to obesity and NCD prevention, corporate governance and food system transformations in Canada and globally.

## Supplementary Information

**Additional file 1: Table A1.** Number of companies with ≥1% market share over 10 years calculated using both national brand owner (NBO) and global brand owner (GBO) market share data in Canada by sector and market. **Table A2.** Four-firm concentration ratios (CR4) over 10 years calculated using both national brand owner (NBO) and global brand owner (GBO) market share data for firms with ≥1% market share in Canada by market. **Table A3.** Herfindahl-Hirschman Index (HHI) over 10 years calculated using both national brand owner (NBO) and global brand owner (GBO) market share data for firms with ≥1% market share in Canada by market.

## Data Availability

Financial and market share data supporting the findings of this study are available from databases which are under license and require paid subscription (24, 26).

## Abbreviations

CR4: Four-firm concentration ratio
GBO: Global Brand Owner
HHI: Herfindahl–Hirschman Index
INFORMAS: International Network for Food and Obesity/non-communicable disease Research, Monitoring and Action Support
NBO: National Brand Owner
NCD: Non-communicable diseases
RTD: Ready-to-drink
