# Supplementary material for: The structure of the Canadian packaged food and non-alcoholic beverage manufacturing and grocery retailing sectors through a public health lens
Source: Global Health. 2023 Mar 11;19:18. doi: 10.1186/s12992-023-00917-w (PMC10008568; doi:10.1186/s12992-023-00917-w)
Supplement: Supplementary file 1 — Additional file 1: Table A1. Number of companies with ≥1% market share over 10 years calculated using both national brand owner (NBO) and global brand owner (GBO) market share data in Canada by sector and market. Table A2. Four-firm concentration ratios (CR4) over 10 years calculated using both national brand owner (NBO) and global brand owner (GBO) market share data for firms with ≥1% market share in Canada by market. Table A3. Herfindahl-Hirschman Index (HHI) over 10 years calculated using both national brand owner (NBO) and global brand owner (GBO) market share data for firms with ≥1% market share in Canada by market. [file 12992_2023_917_MOESM1_ESM.docx]

**Table A1.** Number of companies with ≥1% market share over 10 years calculated using both national brand owner (NBO) and global brand owner (GBO) market share data in Canada by sector and market

|  | **Number of NBOs** | | | | | | | | | | | | | **Number of GBOs** | | | | | | | | | | | | |
| --- | --- | --- | --- | --- | --- | --- | --- | --- | --- | --- | --- | --- | --- | --- | --- | --- | --- | --- | --- | --- | --- | --- | --- | --- | --- | --- |
|  | **2012** | **2013** | **2014** | **2015** | **2016** | **2017** | **2018** | **2019** | **2020** | **2021** | **Mean** | **SD** | **2012** | | **2013** | **2014** | **2015** | **2016** | **2017** | **2018** | **2019** | **2020** | **2021** | **Mean** | **SD** |  |
| **Packaged food manufacturing** | - | - | - | - | 18 | 18 | 17 | 17 | 19 | - | 18 | **1** | - | | - | - | - | 17 | 17 | 18 | 17 | 19 | - | 18 | **1** |  |
| Ready meals | 17 | 17 | 17 | 15 | 15 | 15 | 16 | 17 | 17 | 17 | 16 | 1 | 14 | | 14 | 14 | 12 | 12 | 12 | 12 | 12 | 12 | 12 | 13 | 1 |  |
| Sauces, dressings and condiments | 16 | 15 | 16 | 15 | 15 | 17 | 18 | 18 | 17 | 17 | 16 | 1 | 14 | | 13 | 14 | 13 | 13 | 14 | 14 | 14 | 13 | 13 | 14 | 1 |  |
| Soups | 7 | 6 | 6 | 6 | 7 | 7 | 7 | 9 | 8 | 9 | 7 | 1 | 4 | | 3 | 3 | 3 | 4 | 4 | 4 | 6 | 5 | 6 | 4 | 1 |  |
| Sweet spreads | 10 | 10 | 10 | 11 | 11 | 10 | 11 | 11 | 11 | 11 | 11 | 1 | 8 | | 8 | 8 | 8 | 8 | 7 | 8 | 8 | 8 | 8 | 8 | 0 |  |
| Dairy | 11 | 12 | 12 | 12 | 12 | 11 | 11 | 12 | 12 | 12 | 12 | 0 | 8 | | 9 | 9 | 9 | 9 | 7 | 7 | 8 | 8 | 8 | 8 | 1 |  |
| Confectionery | 11 | 11 | 11 | 11 | 11 | 11 | 10 | 10 | 10 | 10 | 11 | 1 | 10 | | 10 | 10 | 10 | 10 | 10 | 10 | 10 | 10 | 10 | 10 | 0 |  |
| Ice cream and frozen desserts | 5 | 5 | 5 | 5 | 5 | 5 | 7 | 6 | 6 | 6 | 6 | 1 | 5 | | 5 | 5 | 5 | 5 | 5 | 7 | 7 | 7 | 7 | 6 | 1 |  |
| Savory snacks | 12 | 13 | 13 | 13 | 13 | 13 | 13 | 13 | 13 | 15 | 13 | 1 | 10 | | 11 | 11 | 11 | 11 | 12 | 12 | 12 | 12 | 12 | 11 | 1 |  |
| Sweet biscuits, snack bars and fruit snacks | 12 | 12 | 12 | 12 | 12 | 13 | 13 | 13 | 13 | 14 | 13 | 1 | 10 | | 10 | 10 | 10 | 10 | 11 | 11 | 11 | 11 | 12 | 11 | 1 |  |
| Baked goods | 6 | 6 | 6 | 6 | 6 | 6 | 6 | 6 | 7 | 6 | 6 | 0 | 4 | | 4 | 4 | 3 | 3 | 3 | 3 | 3 | 3 | 3 | 3 | 0 |  |
| Breakfast cereals | 6 | 6 | 6 | 6 | 6 | 6 | 6 | 6 | 6 | 6 | 6 | 0 | 4 | | 4 | 4 | 4 | 4 | 4 | 4 | 4 | 4 | 4 | 4 | 0 |  |
| Processed fruits and vegetables | 12 | 12 | 12 | 12 | 12 | 12 | 11 | 11 | 11 | 11 | 12 | 1 | 8 | | 8 | 8 | 8 | 8 | 8 | 8 | 8 | 8 | 8 | 8 | 0 |  |
| Processed meats, seafood and alternatives | 13 | 13 | 13 | 13 | 12 | 12 | 13 | 13 | 13 | 13 | 13 | 0 | 9 | | 9 | 9 | 9 | 9 | 9 | 10 | 10 | 10 | 10 | 9 | 1 |  |
| Rice, pasta and noodles | 16 | 14 | 13 | 13 | 13 | 13 | 13 | 12 | 12 | 13 | 13 | 1 | 13 | | 12 | 11 | 11 | 11 | 11 | 11 | 10 | 10 | 10 | 11 | 1 |  |
| **Non-alcoholic beverage manufacturing** | 14 | 14 | 14 | 14 | 14 | 14 | 14 | 14 | 13 | 13 | 14 | **0** | 11 | | 14 | 13 | 12 | 12 | 12 | 12 | 12 | 11 | 10 | 12 | **1** |  |
| Bottled water | 8 | 8 | 8 | 8 | 8 | 8 | 8 | 9 | 9 | 9 | 8 | 0 | 7 | | 7 | 7 | 7 | 7 | 7 | 7 | 8 | 8 | 8 | 7 | 0 |  |
| Carbonates | 7 | 7 | 7 | 7 | 7 | 7 | 7 | 7 | 7 | 7 | 7 | 0 | 5 | | 5 | 5 | 5 | 5 | 5 | 5 | 5 | 5 | 5 | 5 | 0 |  |
| Concentrates | 5 | 5 | 5 | 5 | 6 | 6 | 6 | 6 | 4 | 4 | 5 | 1 | 5 | | 5 | 5 | 5 | 5 | 5 | 4 | 4 | 4 | 3 | 5 | 1 |  |
| Energy drinks | 7 | 6 | 6 | 6 | 6 | 6 | 5 | 5 | 5 | 4 | 6 | 1 | 8 | | 6 | 6 | 6 | 6 | 6 | 5 | 6 | 6 | 6 | 6 | 1 |  |
| Fruit and vegetable juice | 12 | 12 | 12 | 12 | 12 | 12 | 12 | 11 | 11 | 11 | 12 | 0 | 12 | | 12 | 12 | 10 | 10 | 9 | 9 | 9 | 8 | 7 | 10 | 2 |  |
| RTD coffee | 1 | 2 | 3 | 4 | 6 | 6 | 5 | 5 | 5 | 5 | 4 | 2 | 1 | | 2 | 3 | 4 | 6 | 6 | 5 | 5 | 5 | 5 | 4 | 2 |  |
| RTD tea | 7 | 7 | 7 | 8 | 10 | 11 | 11 | 11 | 10 | 10 | 9 | 2 | 5 | | 5 | 5 | 6 | 9 | 11 | 11 | 11 | 10 | 10 | 8 | 3 |  |
| Sports drinks | 2 | 2 | 2 | 2 | 2 | 2 | 2 | 2 | 2 | 2 | 2 | 0 | 2 | | 2 | 2 | 2 | 2 | 2 | 2 | 2 | 2 | 2 | 2 | 0 |  |
| **Modern grocery retailing** | 7 | 6 | 6 | 6 | 6 | 6 | 6 | 6 | 6 | 6 | 6 | **0** | 7 | | 6 | 6 | 6 | 6 | 6 | 6 | 6 | 6 | 6 | 6 | **0** |  |
| Hypermarkets | 2 | 2 | 2 | 2 | 2 | 2 | 2 | 2 | 2 | 2 | 2 | 0 | 2 | | 2 | 2 | 2 | 2 | 2 | 2 | 2 | 2 | 2 | 2 | 0 |  |
| Supermarkets | 4 | 4 | 4 | 4 | 4 | 4 | 4 | 4 | 4 | 4 | 4 | 0 | 4 | | 4 | 4 | 4 | 4 | 4 | 4 | 4 | 4 | 4 | 4 | 0 |  |
| Discounters | 4 | 4 | 4 | 4 | 4 | 4 | 4 | 4 | 4 | 4 | 4 | 0 | 4 | | 4 | 4 | 4 | 4 | 4 | 4 | 4 | 4 | 4 | 4 | 0 |  |
| Forecourt retailers | 10 | 10 | 10 | 10 | 9 | 8 | 8 | 8 | 8 | 8 | 9 | 1 | 10 | | 10 | 10 | 10 | 9 | 8 | 8 | 8 | 8 | 8 | 9 | 1 |  |
| Convenience | 3 | 3 | 3 | 3 | 3 | 3 | 3 | 3 | 3 | 3 | 3 | 0 | 3 | | 3 | 3 | 3 | 3 | 3 | 3 | 3 | 3 | 3 | 3 | 0 |  |

^1^ As per Euromonitor, national brand owner (NBO) data refers to the producer or distributor of a brand at the national level, whereas global brand owner (GBO) data refers to the company that ultimately owns a brand at the global level (reference: Euromonitor International. Frequently Asked Questions. 2022. Available from: http://www.portal.euromonitor.com).

**Table A2.** Four-firm concentration ratios (CR4) over 10 years calculated using both national brand owner (NBO) and global brand owner (GBO) market share data for firms with ≥1% market share in Canada by market

|  | **CR4 using NBO market share data** | | | | | | | | | | | | **CR4 using GBO market share data** | | | | | | | | | | | | |
| --- | --- | --- | --- | --- | --- | --- | --- | --- | --- | --- | --- | --- | --- | --- | --- | --- | --- | --- | --- | --- | --- | --- | --- | --- | --- |
|  | **2012** | **2013** | **2014** | **2015** | **2016** | **2017** | **2018** | **2019** | **2020** | **2021** | **Mean** | **SD** | **2012** | **2013** | **2014** | **2015** | **2016** | **2017** | **2018** | **2019** | **2020** | **2021** | **Mean** | **SD** |  |
| **Packaged food manufacturing** | | | | | | | | | | | | | | | | | | | | | | | | |  |
| Ready meals | 36 | 35 | 35 | 39 | 38 | 38 | 36 | 35 | 35 | 34 | 36 | 2 | 32 | 32 | 32 | 36 | 35 | 35 | 35 | 33 | 35 | 33 | 34 | 2 |  |
| Sauces, dressings and condiments | 44 | 44 | 43 | 48 | 48 | 48 | 48 | 48 | 49 | 49 | 47 | 2 | 44 | 44 | 43 | 48 | 49 | 51 | 51 | 51 | 52 | 52 | 48 | 3 |  |
| Soups | 82 | 83 | 83 | 83 | 83 | 82 | 82 | 80 | 81 | 81 | 82 | 1 | 78 | 79 | 79 | 79 | 78 | 78 | 78 | 77 | 78 | 78 | 78 | 1 |  |
| Sweet spreads | 51 | 51 | 51 | 49 | 50 | 52 | 52 | 52 | 52 | 52 | 51 | 1 | 47 | 47 | 47 | 47 | 49 | 51 | 52 | 52 | 53 | 53 | 50 | 3 |  |
| Dairy | 64 | 63 | 63 | 63 | 63 | 63 | 62 | 63 | 63 | 63 | 63 | 1 | 62 | 62 | 61 | 61 | 61 | 64 | 64 | 64 | 65 | 65 | 63 | 2 |  |
| Confectionery | 47 | 46 | 46 | 47 | 47 | 47 | 48 | 47 | 48 | 48 | 47 | 1 | 50 | 50 | 50 | 49 | 49 | 48 | 48 | 48 | 49 | 49 | 49 | 1 |  |
| Ice cream and frozen desserts | 78 | 78 | 79 | 79 | 79 | 79 | 77 | 77 | 77 | 77 | 78 | 1 | 76 | 77 | 77 | 77 | 77 | 77 | 75 | 72 | 70 | 70 | 75 | 3 |  |
| Savory snacks | 54 | 54 | 54 | 54 | 54 | 53 | 53 | 53 | 53 | 52 | 53 | 0 | 55 | 55 | 55 | 55 | 55 | 55 | 55 | 54 | 55 | 54 | 55 | 0 |  |
| Sweet biscuits, snack bars and fruit snacks | 44 | 44 | 44 | 42 | 41 | 40 | 39 | 38 | 39 | 38 | 41 | 3 | 44 | 45 | 43 | 41 | 39 | 37 | 36 | 36 | 36 | 35 | 39 | 4 |  |
| Baked goods | 43 | 43 | 43 | 42 | 42 | 41 | 40 | 40 | 43 | 42 | 42 | 1 | 30 | 31 | 31 | 31 | 30 | 30 | 29 | 29 | 31 | 31 | 30 | 0 |  |
| Breakfast cereals | 77 | 78 | 78 | 77 | 77 | 77 | 77 | 77 | 77 | 77 | 77 | 0 | 77 | 78 | 78 | 77 | 77 | 77 | 77 | 77 | 77 | 77 | 77 | 0 |  |
| Processed fruits and vegetables | 45 | 45 | 44 | 43 | 43 | 43 | 48 | 49 | 50 | 50 | 46 | 3 | 41 | 41 | 40 | 39 | 39 | 39 | 40 | 40 | 41 | 41 | 40 | 1 |  |
| Processed meats. seafood and alternatives | 43 | 42 | 40 | 40 | 40 | 40 | 40 | 40 | 40 | 40 | 40 | 1 | 44 | 43 | 41 | 41 | 41 | 40 | 40 | 40 | 41 | 41 | 41 | 1 |  |
| Rice, pasta and noodles | 45 | 49 | 53 | 53 | 53 | 52 | 52 | 53 | 54 | 52 | 51 | 3 | 47 | 46 | 51 | 50 | 50 | 50 | 49 | 50 | 51 | 49 | 49 | 2 |  |
| **Non-alcoholic beverage manufacturing** | | | | | | | | | | | | | | | | | | | | | | | | |  |
| Bottled water | 64 | 65 | 66 | 65 | 63 | 62 | 62 | 62 | 62 | 62 | 63 | 2 | 57 | 57 | 58 | 57 | 55 | 54 | 55 | 54 | 55 | 54 | 56 | 1 |  |
| Carbonates | 79 | 81 | 81 | 81 | 81 | 80 | 80 | 80 | 80 | 80 | 80 | 1 | 77 | 79 | 79 | 79 | 79 | 79 | 78 | 78 | 78 | 78 | 78 | 1 |  |
| Concentrates | 80 | 82 | 78 | 76 | 74 | 73 | 71 | 70 | 73 | 74 | 75 | 4 | 80 | 82 | 78 | 76 | 75 | 73 | 76 | 75 | 74 | 74 ^1^ | 76 | 3 |  |
| Energy drinks | 81 | 80 | 78 | 78 | 77 | 80 | 82 | 83 | 84 | 84 | 81 | 2 | 79 | 79 | 78 | 78 | 77 | 80 | 82 | 81 | 80 | 82 | 79 | 2 |  |
| Fruit and vegetable juice | 53 | 53 | 52 | 53 | 54 | 55 | 56 | 56 | 57 | 57 | 54 | 2 | 49 | 49 | 50 | 51 | 52 | 53 | 54 | 54 | 60 | 60 | 53 | 4 |  |
| RTD coffee | 82 ^1^ | 52 ^1^ | 43 | 50 | 52 | 57 | 60 | 61 | 63 | 62 | 58 | 10 | 82 ^2^ | 52 ^1^ | 43 ^1^ | 50 | 52 | 57 | 60 | 61 | 63 | 62 | 58 | 11 |  |
| RTD tea | 77 | 76 | 75 | 74 | 72 | 72 | 71 | 71 | 70 | 70 | 73 | 3 | 77 | 76 | 75 | 74 | 72 | 68 | 67 | 66 | 66 | 65 | 71 | 5 |  |
| Sports drinks | 99 ^1^ | 99 ^1^ | 99 ^1^ | 99 ^1^ | 99 ^1^ | 99 ^1^ | 99 ^1^ | 99 ^1^ | 99 ^1^ | 99 ^1^ | 99 | 0 | 98 ^1^ | 99 ^1^ | 99 ^1^ | 98 ^1^ | 98 ^1^ | 98 ^1^ | 98 ^1^ | 99 ^1^ | 99 ^1^ | 99 ^1^ | 98 | 0 |  |
| **Modern grocery retailing** | | | | | | | | | | | | | | | | | | | | | | | | |  |
| Hypermarkets^1^ | 100 ^2^ | 100 ^2^ | 100 ^2^ | 100 ^2^ | 100 ^2^ | 100 ^2^ | 100 ^2^ | 100 ^2^ | 100 ^2^ | 100 ^2^ | 100 | 0 | 100 ^2^ | 100 ^2^ | 100 ^2^ | 100 ^2^ | 100 ^2^ | 100 ^2^ | 100 ^2^ | 100 ^2^ | 100 ^2^ | 100 ^2^ | 100 | 0 |  |
| Supermarkets | 86 | 87 | 86 | 87 | 87 | 86 | 87 | 87 | 86 | 87 | 86 | 0 | 86 | 87 | 86 | 87 | 87 | 86 | 87 | 87 | 86 | 87 | 86 | 0 |  |
| Discounters | 78 | 80 | 82 | 82 | 82 | 82 | 83 | 84 | 84 | 84 | 82 | 2 | 78 | 80 | 82 | 82 | 82 | 82 | 83 | 84 | 84 | 84 | 82 | 2 |  |
| Forecourt retailers | 58 | 56 | 55 | 59 | 61 | 62 | 63 | 63 | 62 | 62 | 60 | 3 | 58 | 56 | 55 | 59 | 61 | 62 | 63 | 63 | 62 | 62 | 60 | 3 |  |
| Convenience | 52 ^1^ | 51 ^1^ | 52 ^1^ | 50 ^1^ | 53 ^1^ | 54 ^1^ | 53 ^1^ | 55 ^1^ | 53 ^1^ | 53 ^1^ | 53 | 1 | 52 ^1^ | 51 ^1^ | 52 ^1^ | 50 ^1^ | 53 ^1^ | 54 ^1^ | 53 ^1^ | 55 ^1^ | 53 ^1^ | 53 ^1^ | 52 | 1 |  |

^
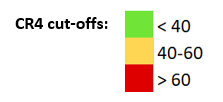
1^ Data available for < 4 companies. ^2^ Less than 4 companies held 100% of the market.

**Table A3.** Herfindahl-Hirschman Index (HHI) over 10 years calculated using both national brand owner (NBO) and global brand owner (GBO) market share data for firms with ≥1% market share in Canada by market

|  | **HHI using NBO market share data** | | | | | | | | | | | | **HHI using GBO market share data** | | | | | | | | | | | |
| --- | --- | --- | --- | --- | --- | --- | --- | --- | --- | --- | --- | --- | --- | --- | --- | --- | --- | --- | --- | --- | --- | --- | --- | --- |
|  | **2012** | **2013** | **2014** | **2015** | **2016** | **2017** | **2018** | **2019** | **2020** | **2021** | **Mean** | **SD** | **2012** | **2013** | **2014** | **2015** | **2016** | **2017** | **2018** | **2019** | **2020** | **2021** | **Mean** | **SD** |
| **Packaged food manufacturing** | | | | | | | | | | | | | | | | | | | | | | | | |
| Ready meals | 507 | 503 | 496 | 523 | 507 | 494 | 470 | 448 | 480 | 458 | 489 | 24 | 452 | 448 | 441 | 468 | 450 | 439 | 436 | 419 | 463 | 447 | 446 | 14 |
| Sauces, dressings and condiments | 622 | 618 | 595 | 938 | 937 | 930 | 934 | 940 | 988 | 995 | 850 | 166 | 620 | 616 | 593 | 932 | 932 | 974 | 984 | 988 | 1044 | 1049 | 873 | 186 |
| Soups | 3805 | 3889 | 3879 | 3865 | 3829 | 3790 | 3754 | 3619 | 3710 | 3700 | 3784 | 88 | 3768 | 3853 | 3844 | 3832 | 3794 | 3755 | 3719 | 3586 | 3677 | 3668 | 3749 | 87 |
| Sweet spreads | 847 | 858 | 842 | 788 | 794 | 826 | 830 | 852 | 867 | 869 | 837 | 28 | 710 | 718 | 705 | 693 | 756 | 786 | 778 | 802 | 814 | 816 | 758 | 48 |
| Dairy | 1203 | 1173 | 1155 | 1141 | 1130 | 1146 | 1142 | 1188 | 1191 | 1205 | 1167 | 28 | 1144 | 1127 | 1103 | 1092 | 1081 | 1198 | 1197 | 1238 | 1242 | 1252 | 1167 | 66 |
| Confectionery | 672 | 650 | 645 | 649 | 641 | 649 | 713 | 710 | 719 | 719 | 677 | 34 | 792 | 767 | 771 | 768 | 756 | 757 | 750 | 745 | 755 | 756 | 762 | 13 |
| Ice cream and frozen desserts | 2449 | 2461 | 2478 | 2470 | 2462 | 2460 | 2326 | 2189 | 2083 | 2041 | 2342 | 173 | 1954 | 1956 | 1971 | 1953 | 1936 | 1929 | 1819 | 1716 | 1639 | 1618 | 1849 | 141 |
| Savory snacks | 1533 | 1540 | 1539 | 1561 | 1558 | 1529 | 1522 | 1506 | 1503 | 1467 | 1526 | 28 | 1581 | 1592 | 1598 | 1611 | 1608 | 1580 | 1573 | 1557 | 1554 | 1518 | 1577 | 28 |
| Sweet biscuits, snack bars and fruit snacks | 614 | 628 | 607 | 570 | 541 | 509 | 490 | 469 | 474 | 463 | 536 | 64 | 562 | 569 | 549 | 507 | 472 | 438 | 416 | 397 | 403 | 393 | 471 | 71 |
| Baked goods | 488 | 501 | 500 | 483 | 471 | 458 | 434 | 425 | 495 | 473 | 473 | 27 | 325 | 333 | 335 | 399 | 385 | 381 | 361 | 351 | 404 | 392 | 367 | 29 |
| Breakfast cereals | 2168 | 2203 | 2155 | 2118 | 2095 | 2076 | 2047 | 2009 | 2024 | 2005 | 2090 | 70 | 2146 | 2179 | 2130 | 2092 | 2068 | 2048 | 2018 | 1979 | 1995 | 1976 | 2063 | 73 |
| Processed fruits and vegetables | 602 | 594 | 577 | 568 | 569 | 567 | 683 | 701 | 724 | 721 | 630 | 68 | 509 | 503 | 488 | 481 | 475 | 475 | 481 | 490 | 514 | 510 | 492 | 15 |
| Processed meats, seafood, and alternatives | 580 | 543 | 496 | 496 | 495 | 493 | 489 | 495 | 519 | 511 | 512 | 29 | 896 | 831 | 752 | 750 | 748 | 743 | 738 | 755 | 802 | 789 | 780 | 51 |
| Rice, pasta, and noodles | 731 | 887 | 1112 | 1082 | 1074 | 1053 | 1029 | 1099 | 1136 | 1017 | 1022 | 124 | 837 | 810 | 1038 | 1010 | 1003 | 984 | 962 | 1035 | 1076 | 714 | 947 | 119 |
| **Non-alcoholic beverage manufacturing** | | | | | | | | | | | | | | | | | | | | | | | | |
| Bottled water | 1501 | 1572 | 1596 | 1563 | 1498 | 1470 | 1459 | 1418 | 1402 | 1364 | 1484 | 77 | 1338 | 1406 | 1425 | 1394 | 1332 | 1313 | 1307 | 1274 | 1264 | 1232 | 1328 | 64 |
| Carbonates | 2184 | 2254 | 2239 | 2206 | 2185 | 2163 | 2122 | 2108 | 2105 | 2067 | 2163 | 62 | 2169 | 2239 | 2224 | 2191 | 2170 | 2148 | 2108 | 2095 | 2091 | 2054 | 2149 | 61 |
| Concentrates | 2642 | 2932 | 2625 | 2546 | 2488 | 2399 | 2287 | 2213 | 2351 | 2262 | 2474 | 220 | 2642 | 2932 | 2625 | 2546 | 2500 | 2411 | 2378 | 2304 | 2442 | 2392 | 2517 | 182 |
| Energy drinks | 2520 | 2300 | 2152 | 2160 | 2155 | 2212 | 2301 | 2308 | 2349 | 2440 | 2290 | 125 | 2438 | 2276 | 2133 | 2160 | 2155 | 2212 | 2301 | 2208 | 2153 | 2152 | 2219 | 96 |
| Fruit and vegetable juice | 887 | 880 | 850 | 860 | 894 | 916 | 945 | 956 | 986 | 999 | 917 | 52 | 743 | 753 | 763 | 781 | 806 | 833 | 860 | 876 | 1097 | 1115 | 862 | 136 |
| RTD coffee | 6740 | 2542 | 1279 | 1573 | 1431 | 1261 | 1338 | 1394 | 1476 | 1462 | 2050 | 1689 | 6740 | 2542 | 1279 | 1573 | 1431 | 1261 | 1338 | 1394 | 1476 | 1462 | 2050 | 1689 |
| RTD tea | 2555 | 2487 | 2470 | 2419 | 2285 | 2078 | 2019 | 1993 | 1967 | 1923 | 2220 | 248 | 2533 | 2467 | 2451 | 2402 | 2270 | 2014 | 1954 | 1927 | 1906 | 1863 | 2179 | 270 |
| Sports drinks | 6570 | 6594 | 6578 | 6531 | 6539 | 6574 | 6432 | 6471 | 6494 | 6483 | 6526 | 54 | 6570 | 6594 | 6578 | 6531 | 6539 | 6574 | 6432 | 6471 | 6494 | 6483 | 6526 | 54 |
| **Modern grocery retail** | | | | | | | | | | | | | | | | | | | | | | | | |
| Hypermarkets | 5212 | 5426 | 5634 | 5800 | 5959 | 6086 | 5977 | 5995 | 5959 | 5959 | 5801 | 287 | 5212 | 5426 | 5634 | 5800 | 5959 | 6086 | 5977 | 5995 | 5959 | 5959 | 5801 | 287 |
| Supermarkets | 2201 | 3381 | 3285 | 3262 | 3263 | 3205 | 3259 | 3222 | 3171 | 3219 | 3147 | 337 | 2201 | 3381 | 3285 | 3262 | 3263 | 3205 | 3259 | 3222 | 3171 | 3219 | 3147 | 337 |
| Discounters | 2655 | 2712 | 2663 | 2616 | 2512 | 2441 | 2418 | 2419 | 2406 | 2405 | 2525 | 124 | 2655 | 2712 | 2663 | 2616 | 2512 | 2441 | 2418 | 2419 | 2406 | 2405 | 2525 | 124 |
| Forecourt retailers | 1442 | 1380 | 1392 | 1755 | 1736 | 1784 | 1777 | 1790 | 1748 | 1728 | 1653 | 173 | 1442 | 1380 | 1392 | 1755 | 1736 | 1855 | 1838 | 1860 | 1817 | 1787 | 1686 | 199 |
| Convenience | 1271 | 1246 | 1309 | 1304 | 1521 | 1609 | 1598 | 1690 | 1611 | 1576 | 1474 | 170 | 1271 | 1246 | 1309 | 1304 | 1521 | 1609 | 1598 | 1690 | 1611 | 1576 | 1474 | 170 |


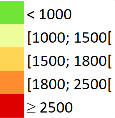


**HHI cut-offs:**
